# Population genetic structure analysis and identification of backfat thickness loci of Chinese synthetic Yunan pigs

**DOI:** 10.3389/fgene.2022.1039838

**Published:** 2022-11-09

**Authors:** Ruimin Qiao, Menghao Zhang, Ben Zhang, Xinjian Li, Xuelei Han, Kejun Wang, Xiuling Li, Feng Yang, Panyang Hu

**Affiliations:** College of Animal Science and Technology, Henan Agricultural University, Zhengzhou, China

**Keywords:** synthetic pig breed, genetic diversity, population structure, selection signature, GWAS, backfat thickness

## Abstract

Yunan is a crossed lean meat pig breed in China. Backfat thickness is the gold standard for carcass quality grading. However, over 14 years after breed registration, the backfat of Yunan thickened and the consistency of backfat thickness decreased. Meanwhile, no genetic study has been ever performed on Yunan population. So, in this study we collected all the 120 nucleus individuals of Yunan and recorded six backfat traits of them, carried out population genetic structure analysis, selection signals analysis and genome-wide association study of Yunan pigs with the help of their founder population Duroc and Chinese native Huainan pigs, to determine the genomic loci on backfat of Yunan. Genetic diversity indexes suggested Yunan pigs had no inbreeding risk while population genetic structure showed they had few molecular pedigrees and were stratified. A total of 71 common selection signals affecting growth and fat deposition were detected by F_*ST*_ and XP-CLR methods. 34 significant loci associated with six backfat traits were detected, among which a 1.40 Mb region on SSC4 (20.03–21.43 Mb) were outstanding as the strong region underlying backfat. This region was common with the results of selection signature analysis, former reported QTLs for backfat and was common for different kinds of backfat traits at different development stage. *ENPP2*, *EXT1* and *SLC30A8* genes around were fat deposition related genes and were of Huainan pig’s origin, among which Type 2 diabetes related gene *SLC30A8* was the most reasonable for being in a 193.21 Kb haplotype block of the 1.40 Mb region. Our results had application value for conservation, mating and breeding improvement of backfat thickness of Yunan pigs and provided evidence for a human function gene might be reproduced in pigs.

## Introduction

Huainan is one of the oldest northern China native pig breeds. It is native to south of the upper reaches of Huai River and North of Dabie Mountains in Henan Province. Henan is located in the middle and lower reaches of the Yellow River and is one of the earliest pig domestication areas in China (Zhang et al., 2007). Huainan is an all-black pig breed with large body size, large ears, short mouth, strong and robust limbs, high fertility, strong adaptability and delicious meat quality (Wang et al., 2005). People in and around Huainan pig producing areas have the habit of eating black pigs. Black pig is an essential ingredient of the famous local cuisine there. However, the poor growth rate of Huainan pigs limited its development.

**FIGURE 1:**
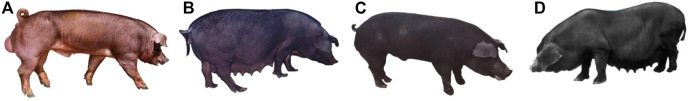
Yunan, Huainan, and Duroc used in this study. **(A)** Duroc. **(B,C)** Yunan **(D)** Huainan.

Since 1980s, a large number of foreign commercial pigs with fast growth rate and high lean meat rate have been introduced to China to improve growth performance of Chinese native pig breeds (Xu, 2004). Among them, the American Duroc with golden coat color was widely used because of its high growth rate and high feed conversion rate (Figure 1A). So, in 1996, we started to use Huainan and American Duroc pigs as the base herd to intercross to develop a new high-performance black pig, Yunan pig (Figures 1B,C) (Zhu et al., 2009).

**FIGURE 2:**
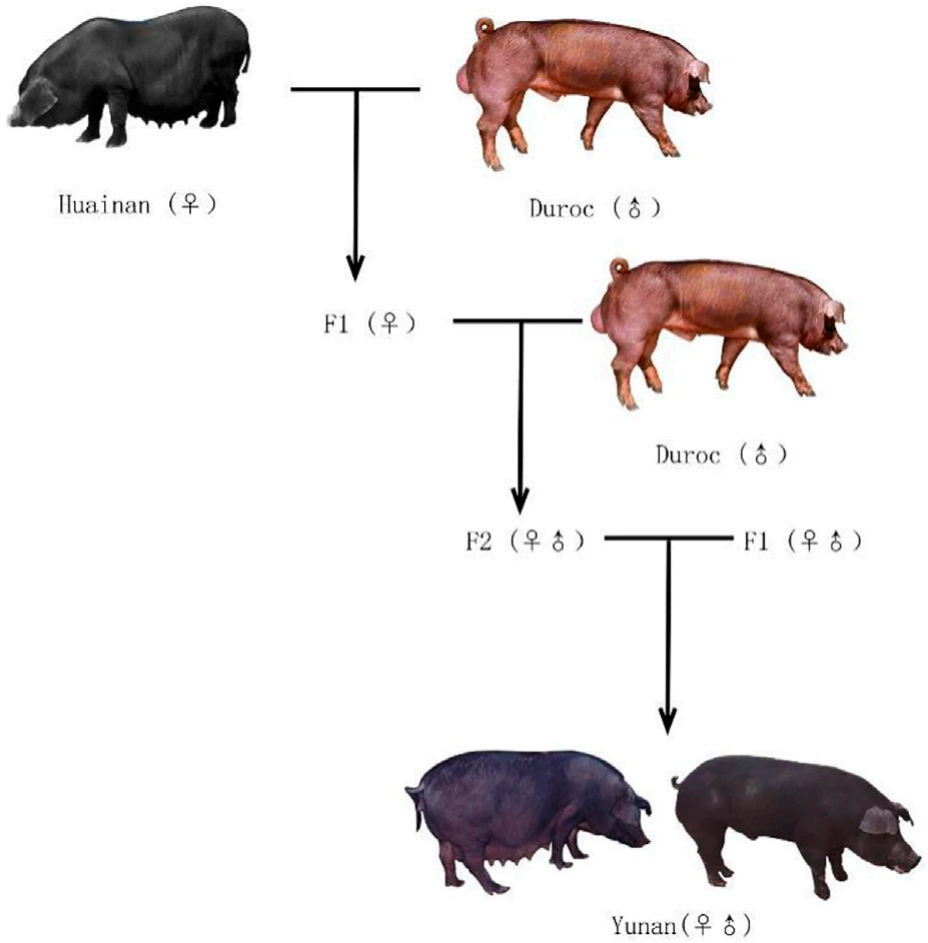
Schematic representation of the setup to generate Yunan using Huainan and Duroc.

Yunan pig is a black crossbred pig breed obtained by nine generations crossbreeding between Huainan and American Duroc (Figure 2). In 2008, Yunan was registered as a new pig breed in China by National Livestock and Poultry Genetic Resources Committee. Theoretically, it contains 37.50% of Huainan’s lineage and 62.50% of Duroc lineage. Yunan exhibits good growth performances from Duroc with an average daily gain of 648 g during the stage of body weight at 30 kg–90 kg and lean meat rate of 56%, and has good meat quality from Huainan with an excellent meat quality (intramuscular fat content as 4.11%). So, Yunan becomes a popular black pig breed in and around Henan.

Since the year of 2008, the systematic breeding of Yunan pigs have been no longer carried out which resulted in a decline of backfat consistency. Backfat thickness now is still the only gold standard for black pig carcass grading as same as the commercial pigs in China. Therefore, the decline of backfat performance of Yunan brings great economic loss to farmers. Meanwhile, with the emergence of the African swine fever in China in 2018, the population of Yunan pigs reduced seriously.

However, there has never been a genomic study on Yunan pigs. Only a few correlation analyses of several genes and comparative analysis of production traits of Yunan were reported (Li et al., 2016; Wang et al., 2021). Herein, in this study, we recorded six backfat thickness traits of the core group of Yunan population, and used SNP array genotyping data of Yunan, Huainan and Duroc with the following objectives: 1) determine the genetic diversity of Yunan by calculating the observed heterozygosity and the expected heterozygosity; 2) access the inbreeding state by detecting runs of homozygosity (ROH); 3) detect selection signals of Yunan by pairwise F_*ST*_ and XP-CLR methods; 4) investigate genomic evidence on the population structure of Yunan by phylogenetic tree, principal component and admixture analysis; 5) identify the genomic region that controlled backfat thickness of Yunan.

## Material and methods

### Animals sampling, genotyping and phenotyping

Ear tissues were collected from Yunan (*n* = 120) and Huainan (*n* = 33) pigs at Sungo Agricultural and Livestock Co. Henan, China. Genomic DNA was extracted using the phenol-chloroform method and genotyped by 50 K SNP (Compass) and 80 K SNP (NEOGEN). Five backfat thickness traits including shoulder backfat (SBF), sixth and seventh ribs backfat (SSRBF), last rib backfat (LRBF), lumbar joint backfat (LBF) and P2 backfat (P2BF) were measured and recorded for Yunan pigs using ultrasonic instrument (EXAGO, France). Average backfat (ABF) was calculated as the average of SBF, SSRBF, LBF and LRBF. Genotype data of American Duroc pigs (*n* = 40) (Illumina PorcineSNP60 Genotyping Bead Chip) was downloaded from the public Dryad database (http://dx.doi.org/10.5061/dryad.30tk6).

We used PLINK v.1.9 (Purcell et al., 2007) to perform the quality control (QC) of the total 193 individuals genotype data. SNPs without positions on pig reference genome (*Sus scrofa* 11.1), or with genotyping rate less than 90%, or with minor allele frequency (MAF) lower than 1% or on Y chromosome were excluded. Individuals with genotyping rate less than 90% were excluded. Genotypic data of the same SNP in Yunan, Huainan and Duroc populations were extracted for genetic diversity, genetic differentiation and population genetic structure analyses.

### Genetic diversity and selection signal analysis

Genetic indicators including the observed heterozygosity (Ho), the expected heterozygosity (He) and MAF were calculated for Yunan, Huainan and Duroc by PLINK v.1.9 to compare the genetic diversity of these populations, command set is "-hardy”. In addition, SNePv1.1 (Barbato et al., 2015) software was used to calculate the effective population size (Ne).

The length and frequency of ROH can reflect the group history. A long ROH indicates recent inbreeding, while a short ROH indicates ancient inbreeding. Genomic homozygous fragments of each individual were detected using runs of homozygosity of PLINK v.1.9. The following parameters were used of define ROHs: 1) the minimum length of ROHs was 500 Kb; 2) a sliding window of 50 SNPs across the genome; 3) each window allowed one heterozygous genotype and five missing SNPs (Xu et al., 2021) to avoid false negatives caused by occasional genotyping errors and missing genotypes. Then the ratio of the total length of the ROH fragment to the total length of autosomal genome was calculated to get the coefficient of inbreeding (Silió et al., 2013) using the following formula:

$$F_{ROH}=\frac{\sum L_{ROH}}{L_{auto}},$$ (1)

where $L_{ROH}$ is the sum of the lengths of the autosomal ROH fragments and $L_{auto}$ is the total size of 18 autosomes of pigs covered by SNPs, which is 2.265 Gb (https://www.ncbi.nlm.nih.gov).

We used SNP data from three breeds, using genetic differentiation coefficient (F_*ST*_) and cross-population compound likelihood ratio test (XP-CLR) for genome-wide selective detection. The genetic differentiation coefficient (F_*ST*_) between populations was calculated using Vcftools v.0.1.13 (Sun et al., 2020), The command set was as follows: 1) window size was set to 500 Kb; 2) step length was set to 40 Kb. The F_*ST*_ values ranging from 0 to 0.05, 0.05 to 0.15, and 0.15 to 0.25 indicate that there are no genetic, moderate, and large differentiations among populations, respectively. XP-CLR was calculated using XP-CLR (Chen et al., 2010) software. XP-CLR models the difference in frequency of multiple alleles between the two populations. Further, we used the parameters (“-w1 0.005 200 2000 -p0 0.95”) to calculate the XP-CLR score for each chromosome. The empirical cutoffs for the genomic windows with top 1% F_*ST*_ and XP-CLR values across the whole genome were considered as selective sweeps.

### Population genetic structure analysis

To assess the individual genetic distances between populations to illustrate the relationship between populations, we carried out principal components analysis (PCA) of the SNP dataset using PLINK v.1.9 and selected the first two principal components for visualization by ggplot package in R v.4.1.3 (Team, 2014). To gain more insight into the Yunan pig population, we performed t-distributed stochastic neighbour embedding (T-SNE) analysis on the dataset using the R package “Rtsne” (Krijthe, 2015). The phylogenetic tree was built by neighbor-joining (NJ) method and visualized using R v.4.1.3, the genetic distance matrix was constructed by PLINK v.1.9. To explore the population structure of Yunan, the rapid model of ADMIXTURE v.1.3 (Alexander et al., 2015) software was used to cluster all Yunan samples in a context of some related populations and the results were visialized using the “barplot” package of R v.4.1.3 software.

### Genome-wide association analysis of backfat thickness

Genome-wide association analysis for backfat traits of Yunan was performed using a univariate mixed linear model of GEMMA v.0.98.5 (Zhou et al., 2012) as follows.

y=Wα+xβ+u+ε, (2)

where y is a vector of phenotypic values for the trait; W is a matrix of fixed effects including the first three PCs; α is a vector of corresponding coefficients including the intercept; x is a vector of genotypes for the marker; β is an effect size for the marker; u is an n-vector of random effects; and ε is an n-vector of errors.

The results of genome-wide association analysis were visualized using the rMVP (Yin et al., 2021) package in R software. The suggestive significance threshold was 1/N, where N is the number of SNPs used for the analysis. We used 1 Mb upstream and downstream significant SNPs region as traits related candidate region.

## Results

### Genotyping

There are 57,466 SNPs sites in the original data. After quality control, SNPs without positions on pig reference genome (Sus scrofa 11.1), or with genotyping rate less than 90%, or with minor allele frequency lower than 1% or on Y chromosome were excluded. Individuals with genotyping rate less than 90% were excluded. Finally, 50,717 SNPs were used for the following analysis.

### Genetic diversity analysis

To assess genetic diversity of Yunan, we analyzed MAF, Ho, He and Ne of Yunan, Huainan and Duroc. The results were shown in Table 1. By comparison, the Ne of Huainan was lower than that of Yunan and Duroc. Indexes MAF, Ho and He of Yunan were higher than those of Huainan and Duroc indicating that Yunan was rich in genetic diversity.

**TABLE 1:** Summary of population genetic diversity indexes.

Breed	No	Ne	MAF	H_O_	He
Yunan	120	67	0.2813	0.3756	0.3685
Huainan	33	43	0.2337	0.3422	0.3193
Duroc	40	77	0.2462	0.3019	0.3277

ROH analysis results of Yunan, Huainan and Duroc were shown in Table 2. A total of 3,317 homozygous fragments were detected in the three populations. Duroc had the highest average number of ROH per animal (48.8 ± 6.55 with a range of 2–80 Mb) while Huainan had the lowest (7.21 ± 8.14 with a range of 1–105 Mb). Mean length of ROH (MGL_ROH_) was maximum in Yunan (10.01 ± 4.28 Mb) and minimum in Duroc (8.91 ± 1.14 Mb). Duroc revealed the highest ROH based inbreeding (F_*ROH*_ = 0.1925 ± 0.037). F_*ROH*_ of Huainan (F_*ROH*_ = 0.0324 ± 0.043) and Yunan (F_*ROH*_ = 0.043 ± 0.033) were lower than Duroc. Yunan did not have severe inbreeding.

**TABLE 2:** Genomic distributions and descriptive statistics of ROH in Yunan, Huainan and Duroc.

Breeds	N_ROH_	Range ROH (Mb)	NM_ROH_	MGL_ROH_ (Mb)	F_ROH_
Yunan	1,126	3–124	9.38 ± 4.55	10.01 ± 4.28	0.043 ± 0.033
Huainan	238	1–105	7.21 ± 8.14	9.49 ± 6.21	0.034 ± 0.043
Duroc	1953	2–80	48.82 ± 6.55	8.91 ± 1.14	0.1925 ± 0.037

N_ROH_: Total number of ROH per breed; Range ROH: length range of ROH; NM_ROH_: Mean number of ROH in a breed; MGL_ROH:_ Breed wise mean genome length covered by ROH in Mb; F_ROH:_ Inbreeding coefficient based on ROH

**FIGURE 3:**
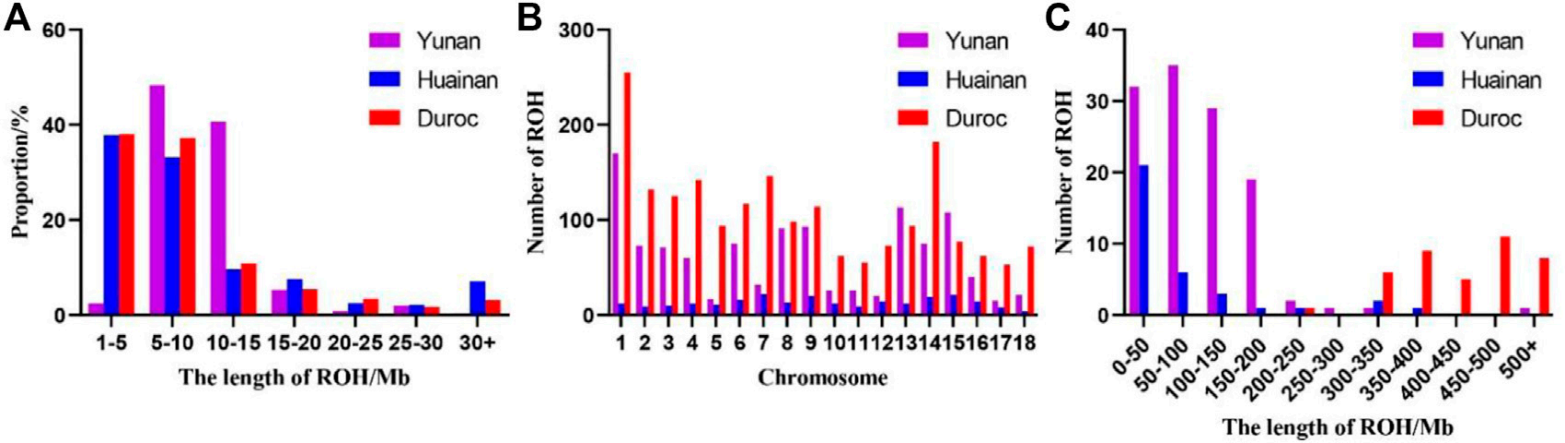
**(A)** Distribution of ROH length in Yunan, Huainan and Duroc. **(B)** Distribution of ROH quantity on Yunan, Huainan and Duroc chromosome. **(C)** Distribution of ROH samples number in Yunan, Huainan and Duroc.

ROH fragment size of Yunan mainly concentrated in 5–15 Mb, accounting for 88.98% while that of Duroc and Huainan mainly concentrated in 1–10 Mb, accounting for 75.32% and 71.01%, respectively (Figure 3A). Chromosomes with the most ROH fragments in Yuanan, Duroc and Huainan were SSC1 (*n* = 170), SSC1 (*n* = 255) and SSC7 (*n* = 22), and chromosomes with the least ROH fragments were SSC17 (*n* = 15), SSC17 (*n* = 53) and SSC18 (*n* = 4) (Figure 3B). The total average length of ROH of Yunan, Duroc and Huainan were 98.37 Mb, 436.28 Mb and 73.32 Mb (Figure 3C).

### Population genetic structure analysis of yunan population

**FIGURE 4:**
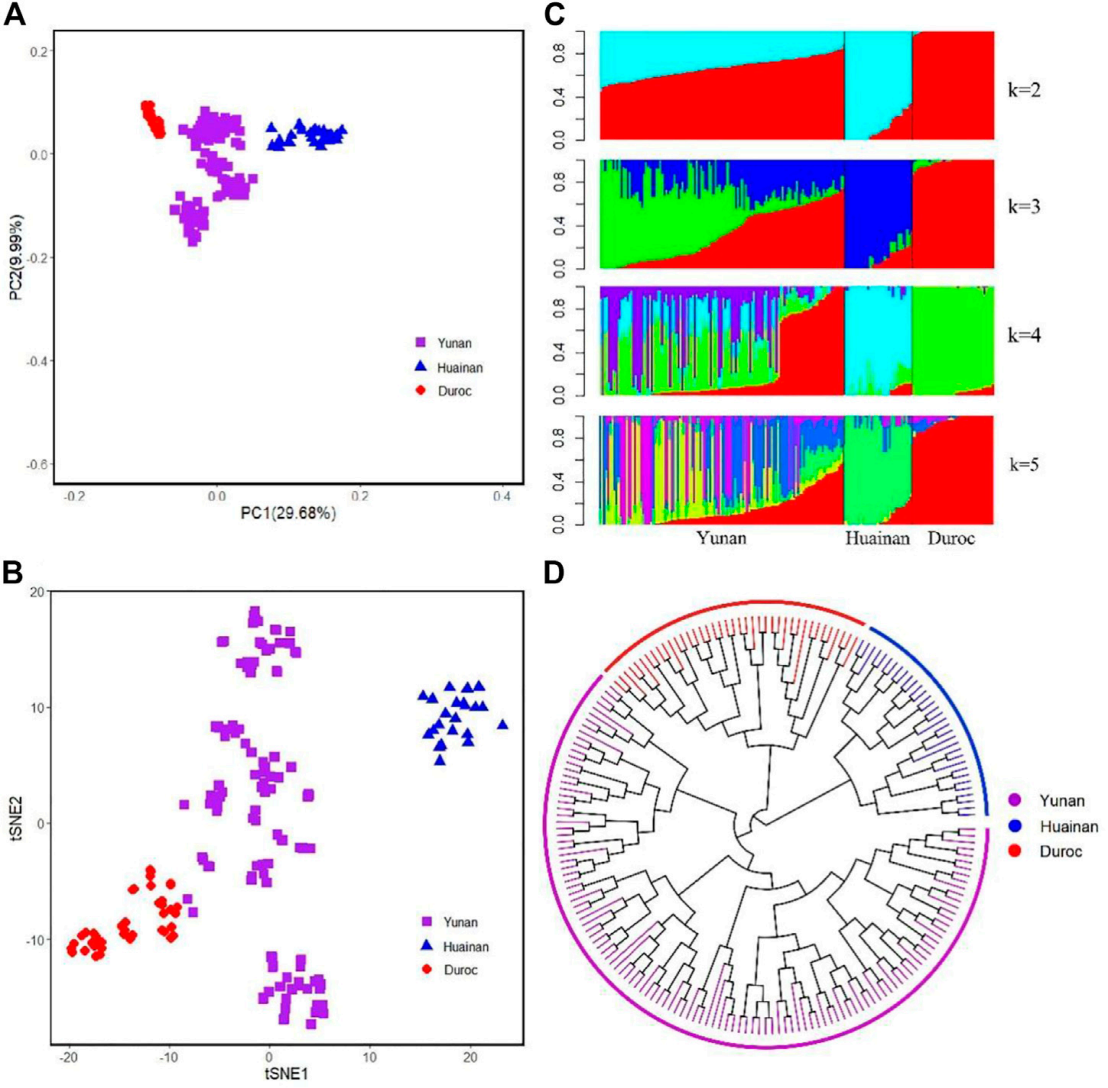
Population structure analyses for all pig individuals. **(A)** First and second principal components from a principal component analysis of all populations. **(B)** t-SNE plot for all breeds. **(C)** Population structure plots for all pig populations at K = 2, 3, 4, and 5. **(D)** Neighbor-joining tree for all individual pigs.

The first and second principal components of Yunan, Huainan and Duroc explained 29.68% and 9.99% of the total variance. Yunan, Huainan, and Duroc were clearly separated (Figure 4A). The first PC clearly separated Yunan, Huainan and duroc. Yunan was located between Huainan and Duroc. We used t-SNE to best classify the populations to perform dimensionality reduction clustering analysis on all the breeds. From Figure 4B, these results indicate that different subpopulations exist in Yunan pig population. From the result of admixture analysis in Figure 4C, When K = 2, Yunan, Huainan and Duroc could be clearly distinguished. Yunan contained 33.78% of Huainan and 66.22% of Duroc blood. When K = 3, a new bloodline in red was appeared in Yunan. When K = 4-5, different degrees of differentiation appeared in Yunan population. To investigate genetic structure of Yunan pigs, we constructed the NJ tree of the 193 individuals, which indicated the existing Yunan pigs could be divided into four main branches, each of which was divided into two or more subbranches (Figure 4D).

### Selection signal screening among yunan, Huainan and Duroc

**FIGURE 5:**
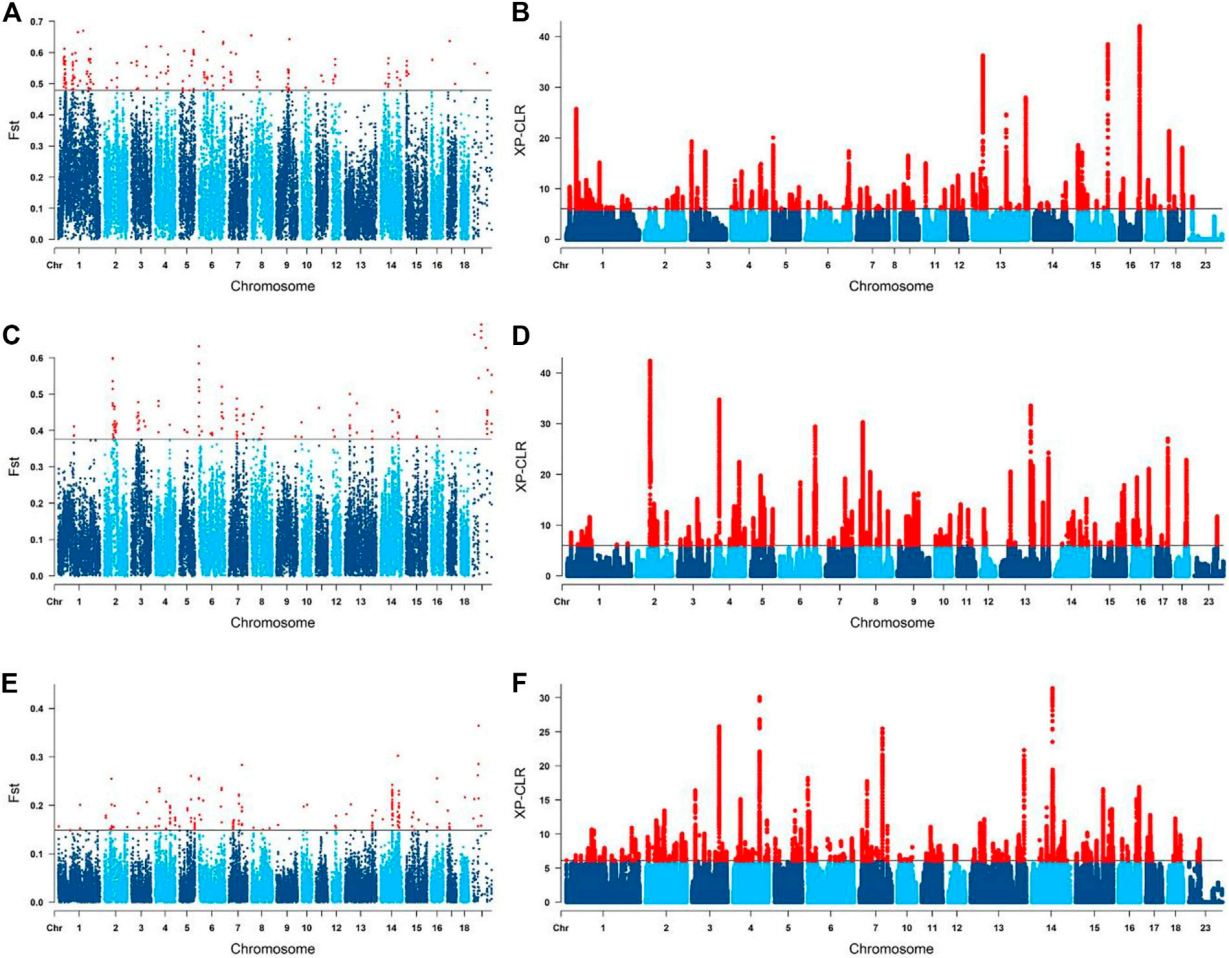
Selected signature detection for different groups on manhattan graphs. **(A,B)** yunan-duroc vs. huainan. **(C,D)** yunan-huainan vs. duroc. **(E,F)** yunan vs. huainan-duroc.

To screen the selection signals among Yunan, Huainan and Duroc, and analyse the possibly origin of the signals, we divided Yunan, Huainan and Duroc into three groups (Yunan-Duroc Vs. Huainan, Yunan-Huainan Vs. Duroc and Yunan Vs. Huainan-Duroc) and used two methods of F_*ST*_ and XP-CLR. Top 1% regions of F_*ST*_ and XP-CLR were considered as salient loci under selected. F_*ST*_ screening results were shown in Figure 5A (Yunan-Duroc Vs. Huainan), **5C** (Yunan-Huainan Vs. Duroc) and **5E** (Yunan Vs. Huainan-Duroc). We detected several significant loci on SSC1-12, SSC14-17 and SSCX in Yunan-Duroc Vs. Yunan group, SSC1-16 and SSCX in Yunan-Huainan Vs. Duroc group, and SSC1-10, SSC12-18 and SSCX in Yunan-Duroc Vs. Yunan group. XP-CLR analysis results of the above three groups were shown in Figure 5B (Yunan-Duroc Vs. Huainan), **5D** (Yunan-Huainan Vs. Duroc) and **5F** (Yunan Vs. Huainan-Duroc). We obtained 71 significant loci on each of the 18 autosomes in each of the three groups.

Then we combined the outputs of F_*ST*_ and XP-CLR analysis. In Yunan-Duroc Vs. Huainan group, we found 37 significant regions that over threshold under selected were overlapped in F_*ST*_ and XP-CLR results. A total of 104 genes were annotated in these regions in the Ensembl (http://ensemble.org) database, 17 out of which were involved in fat production and metabolism, nine out of which were related to growth and development. These 26 genes under selected were thought to be of Duroc origin.

Similarly, in Yunan-Huainan Vs. Duroc group, we got 21 overlapping regions containing 103 annotated genes, 14 genes out of which were related to fat regulation while eight genes were related to growth development. These 22 genes were thought to be of Huainan origin. Also, 13 overlapping regions covering 116 genes were found in Yunan Vs. Huainan-Duroc. Only eight genes were involved in fat metabolism and adipose production, three genes were involved in skeletal muscle development. These outstanding 11 genes may have been selected by artificial selection in Yunan population.

To further look into former reported economic characters underlying these genomic regions, we extracted the common regions between these results and PigQTLdb (www.animalgenome.org/cgi-bin/QTLdb/index). QTLs that overlapped with potentially selected regions were QTLs of average daily gain (Fontanesi et al., 2014; Guo et al., 2017), backfat last rib (Gilbert et al., 2007) and days 110 kg (Wang et al., 2015), etc. as shown in Table 3.

**TABLE 3:** Candidate genes and previous reported QTLs of common selected regions from F_*ST*_ and XP-CLR analysis.

Group	SSC	Position (bp)	Gene		QTL
			Backfat	Growth	
Yunan-Duroc Vs. Huainan	1	44,480,000–4,4,931,794	GPRC6A		
	1	90,160,000–90,495,794		SENP6	
	1	97,080,000–97,995,794	SMAD2, ZBTBZ7C		Body weight (birth)
	3	2,770,524–3,132,524	SDK1	SLC26A2	Body weight (end of test)
	3	36,784,268–37,498,268		C16orf89	
	3	71,302,268–71,340,268	ZNF638		
	6	54,733,496–55,767,496	CPT1	CLEC11A	Average daily gain
	7	14,960,000–15,610,808	ID4		Backfat at last rib
	8	978,400–1036400	NSD2	NELFA	Leaf fat weight
	9	76,400,000–7734,000	TAC1	DLX5	
	9	109,103,272–109,460,000	SFRP5	CRTAC1	
	15	3,800,000–4,580,000		MBD5, ACVR2A	Average backfat thickness
	17	17,009,180–17,211,180	PLCB1		
Yunan-Huainan Vs. Duroc	2	56,423,768–56,919,768	NLRP3		
	2	5,8,071,768–60,123,768	LPAR2, GATAD2A, SUGP1, NCAN, INSL3, JAK3, CRTC1	CLIP2, MEF2B, SLC25A42, CRLF1, GDF15	Body depth
					Body width
	2	68,667,768–68,780,000	PIN1, OLFM2	UBL5	
	2	69,440,001–69,625,768	DNM2, CARM1	TGFBR3L	Backfat at first rib
	4	21,240,001–21,830,268	SLC30A8		Average backfat thickness
	6	149,794,156–150,244,156	ANGPTL3		Body weight
					Days to 100 kg
	13	33,334,864–33,480,864		DOCK3	
Yunan Vs. Huainan-Duroc	2	44,416,175–44,614,028	PDE3B		
	6	4,808,255–5,832,188	CDH13		Body length
	6	29,366,624–29,413,703	AMFR		
	7	28,300,808–29,064,808	BEND6	RAB23	Average daily gain
	12	22,829,286–23,387,286	MED1, PIP4K2B		
	14	75,501,272–77,161,272	MCU, PLA2G12B, ANXA7	MSS51, MYOZ1	
	14	112,560,001–113,175,272	LDB1		

## Genome-wide association study on backfat traits in yunan pigs

To understand genetic background of backfat thickness variation in Yunan, Yunan pigs were divided into gilt (n = 48), 1^st^ parity (n = 48) and 2^nd^ parity (n = 24) according to the age. Descriptive statistics of the six backfat traits (P2BF, SBF, SSRBF, LRBF, LBF and ABF) of this three groups were shown in Table 4. From Table 4, Yunan was much fatter from the ideal body shape. For example, the average P2BF thickness of gilt, 1^st^ parity and 2^nd^ parity in Yunan was 36.90, 35.28 and 35.93 mm. This was much thicker than the commercial sows (usually 16–22 mm). Moreover, the coefficient variation (CV) of the backfat traits was larger, ranged from 12.80% to 34.96%.

**TABLE 4:** Descriptive statistical of backfat traits in Yunan pigs.

	Number	Trait	Mean (mm)	SD	Min	Max	CV %
Gilt	48	P2BF	36.90	9.21	20.70	53.90	34.96
		SBF	15.70	3.24	9.40	26.90	20.64
		SSRBF	40.00	9.29	23.20	58.90	23.23
		LRBF	34.64	8.37	20.10	53.90	24.16
		LBF	20.58	3.24	15.00	27.60	15.74
		ABF	27.70	5.04	19.58	37.93	18.19
Paity 1	48	P2BF	35.28	7.37	20.70	50.80	20.89
		SBF	18.40	2.73	13.80	26.30	14.84
		SSRBF	35.77	8.74	20.70	54.50	24.43
		LRBF	31.96	7.50	18.20	52.60	23.47
		LBF	21.78	3.99	13.80	36.40	18.32
		ABF	26.98	4.79	18.00	39.03	17.75
Paity 2	24	P2BF	35.93	6.74	22.60	52.00	18.76
		SBF	18.36	2.35	13.80	24.40	12.80
		SSRBF	39.67	9.94	21.30	60.30	25.06
		LRBF	32.87	6.54	21.30	50.10	19.90
		LBF	21.76	2.88	17.50	30.10	13.24
		ABF	28.17	5.03	19.43	40.43	17.86

P2BF, P2 point backfat; SBF, Shoulder backfat; SSRBF, Six and seven rib backfat; LRBF, Last rib backfat; LBF, Lumbosacral backfat; ABF, Average backfat; SD, Standard Deviation; CV, Coefficient of variance.

**FIGURE 6:**
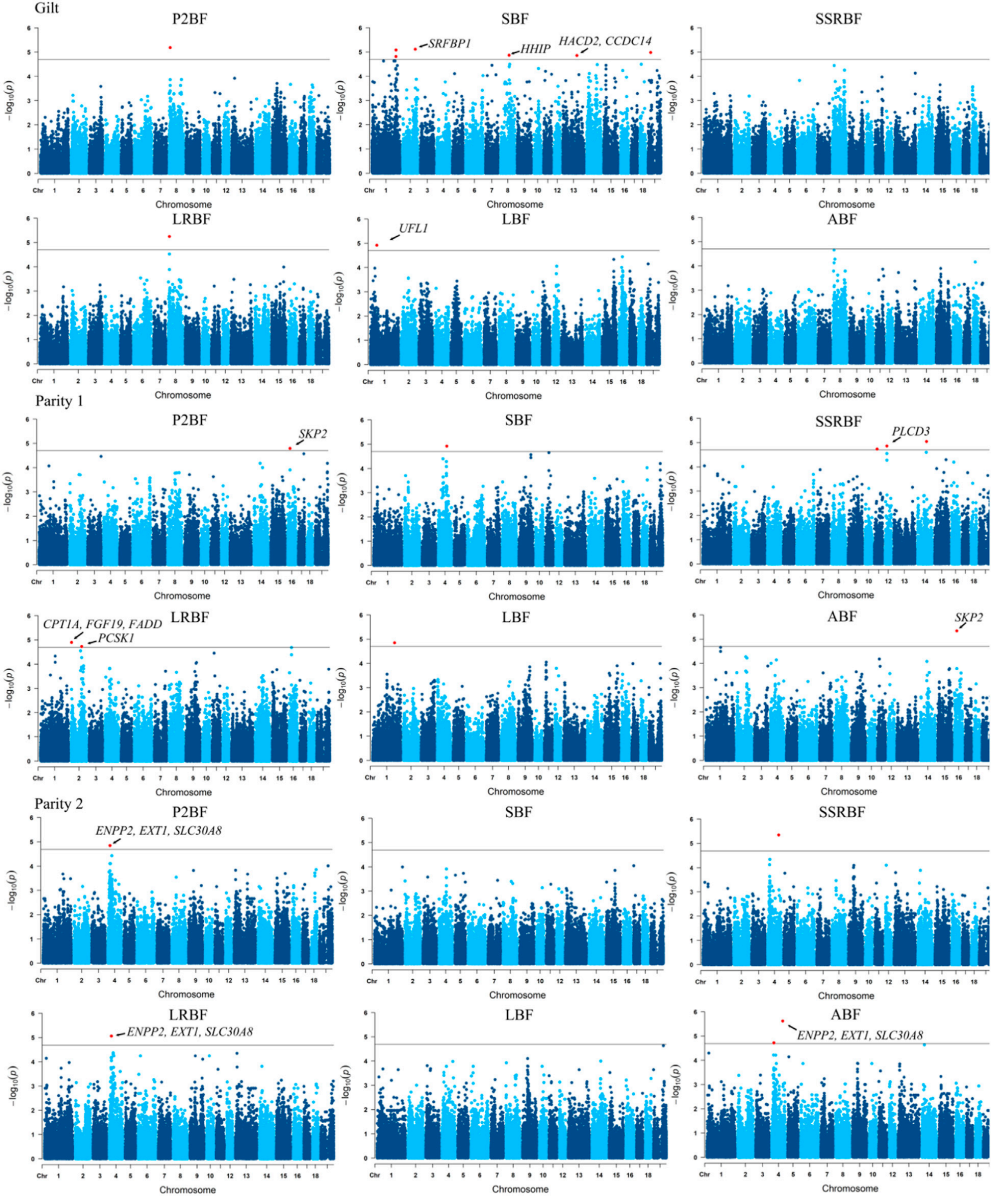
Manhattan plot of genome-wide association analysis of P2-backfat, shoulder backfat, sixth and seventh ribs backfat, last rib backfat, lumbar joint backfat and average backfat at different parity. The annotated genes represent the relevant candidate genes.

With the goal of pinpointing genomic region associated with backfat phenotypes of Yunan, we performed genome-wide association studies of six backfat traits using a linear mixed model. We identified 34 suggestive significant SNPs associated with six backfat traits in total. Thereinto, nine significant SNPs on SSC1-2, SSC4, SSC11-12, SSC14 and SSC16 were detected in 1^st^ parity pigs. 14 significant SNPs on a 1.4 Mb segment of SSC4 (20.03–21.43 Mb) were detected in 2^nd^ parity pigs while 11 significant SNPs on SSC1-2, SSC8, SSC13 and SSCX were obtained in gilts. 202 genes were annotated in the 34 genomic regions in the Ensembl database, five, six and three genes of which were involved in fat metabolism or growth in gilt, 1^st^ and 2^nd^ parity pigs (Figure 6).

The genomic regions where these significant loci were located partially overlapped. The most significant associated SNP was the same for P2BF and LRBF on SSC8 (10.43 Mb) in gilts, for P2BF and ABF on SSC16 (21.97 Mb) in 1^st^ parity, for P2BF, LRBF and ABF on SSC4 (20.03–21.43 Mb) and for SSRBF and ABF on SSC4 (106.29 Mb) in 2^nd^ parity pigs. Then we combined these 34 regions with PigQTLdb database (www.animalgenome.org/cgi-bin/QTLdb/index) and found that significant loci on SSC4 (20.03–21.43 Mb) were overlapped with a reported average backfat thickness QTL (20,366,121–21,945,045 bp) in large white pigs (Bink et al., 2000) (Table 5).

**TABLE 5:** Summary of genomic regions significantly associated with six backfat traits in Yunan pigs.

Parity	Phenotype	SSC	Position (Mb)	Number of significant SNPs	Most significant SNP		Minor allele frequency	Candidate gene name
					Position (bp)	*p*-value		
Gilt	P2BF	8	10.43	1	10,430,709	6.58E-06	0.2396	
	SBF	1	233.79–235.18	4	234,643,280	8.30E-06	0.125	
		2	125.35	1	125,353,968	7.77E-06	0.375	*SRFBP1*
		8	83.12	1	83,125,662	1.37E-05	0.08333	*HHIP*
		13	136.08	1	136,088,769	1.40E-05	0.2812	*HACD2, CCDC14*
		23	23.89	1	23,893,572	1.05E-05	0.05208	
	LRBF	8	10.43	1	10,430,709	5.69E-06	0.2396	
	LBF	1	63.41	1	63,418,505	1.21E-05	0.09375	*UFL1*
Parity 1	P2BF	16	21.97	1	21,976,238	1.61E-05	0.4271	*SKP2*
	SBF	4	83.26	1	83,269,208	1.21E-05	0.4375	
	SSRBF	11	26.09	1	26,093,623	1.84E-05	0.1354	
		12	17.86	1	17,867,041	1.38E-05	0.1562	*PLCD3*
		14	85.98	1	85,986,081	9.06E-06	0.0625	
	LRBF	2	3.52	1	3,528,858	1.27E-05	0.1458	*CPT1A, FGF19, FADD*
		2	102.23	1	102,231,770	1.89E-05	0.1562	*PCSK1*
	LBF	1	213.80	1	213,808,326	1.42E-05	0.01042	
	ABF	16	21.97	1	21,976,238	4.61E-06	0.4271	*SKP2*
Parity 2	P2BF	4	20.03–21.43	4	20,038,718	1.44E-05	0.4792	*ENPP2, EXT1, SLC30A8*
	SSRBF	4	106.29	1	106,293,182	4.47E-06	0.1250	
	LRBF	4	20.03–21.43	4	20,038,718	8.73E-06	0.4792	*ENPP2, EXT1, SLC30A8*
	ABF	4	106.29	1	106,293,182	2.44E-06	0.1250	
		4	20.03–21.43	4	20,038,718	1.91E-05	0.4792	*ENPP2, EXT1, SLC30A8*

Significance of SNP.

In view of the existence of selective signals related to fat deposition and growth, and to investigate whether the genomic regions associated with backfat traits mapped by GWAS might have been selected in the population, we analyzed the regions common to the results of F_*ST*_, XP-CLR and GWAS. The selected region on SSC4 (21.24–21.83 Mb) in Yunan-Huainan Vs. Duroc group which was thought to be of Huainan origin and the P2BF, LRBF and ABF associated region on SSC4 (20.03–21.43 Mb) in 2nd parity stood out. These two regions were overlapped, and *ENPP2* and *EXT1* genes (SSC4:19.02–21.06 Mb) in this region were functionally related to fatty acid production (Crespo-Piazuelo et al., 2020). Therefore, the genome region of 20.03–21.83 Mb on SSC4 was considered as an important candidate region for backfat thickness in Yunan pig population.

**FIGURE 7:**
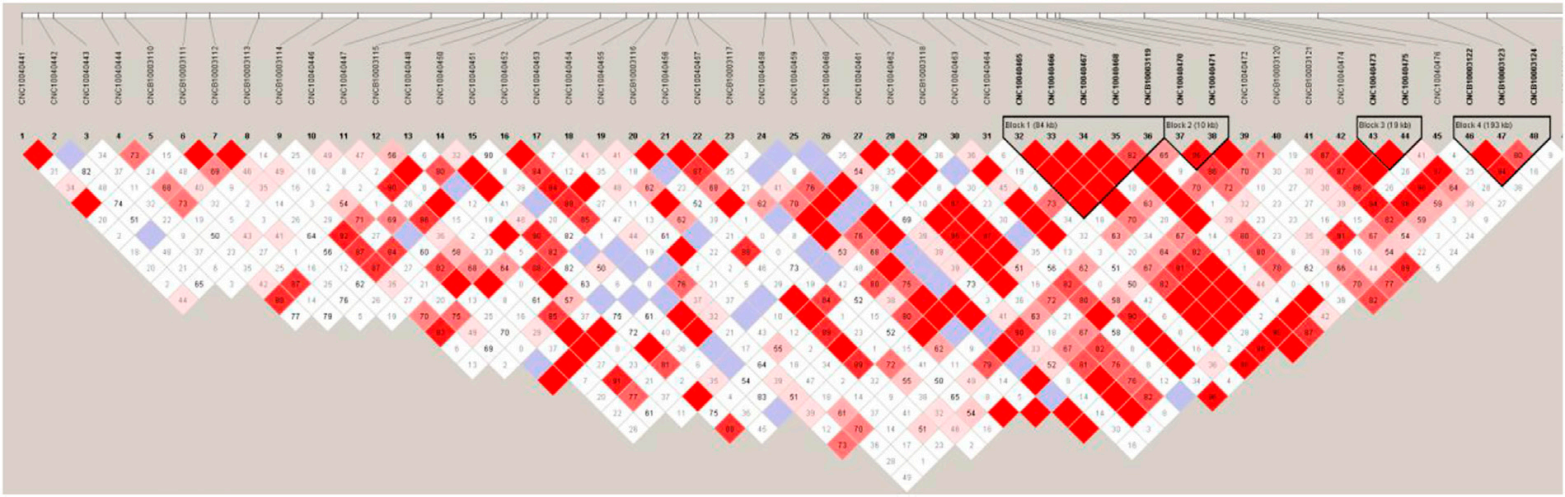
Haploview of linkage disequilibrium of SNPs on chromosome four (20.03–21.83 Mb) in Yunan pigs.

Finally, we used Haploview v4.2 (Barrett et al., 2005) software for linkage disequilibrium analysis and constructed haplotype modules for the SSC4 (20.03–21.83 Mb) under the default parameters. We found four linkage blocks (*r* ^2^≥0.8), block1 (21, 111, 825–21,196,785 bp), block2 (21, 208, 018–21,218,173 bp), block3 (21, 402, 451–21,421,498 bp) and block4 (21, 516, 432–21,709,640 bp) within the target area (Figure 7). Only one gene *SLC30A8* (21, 517, 486–21,555,757 bp) was found in block4. *SLC30A8* gene encodes zinc transporter, which transfers zinc from the cytoplasm of pancreatic β-cells to intracellular vesicles and functions in insulin secretion. Impaired insulin secretion and insulin resistance are the pathogenesis of type 2 diabetes mellitus (Witka et al., 2019).

## Discussion

The genetic diversity of a population was a key factor in ensuring the survival and evolution of a species. Commercial pigs have been subjected to high-intensity artificial selection for a time, resulting in a decrease in genetic diversity, while local varieties were mainly selected by natural conditions such as environmental factors and geographical factors, thus maintaining a high level of genetic diversity. China’s native pig breeds has a better genetically diverse (SanCristobal et al., 2006; Ai et al., 2013). The genetic diversity of Chinese hybrid pigs was higher than that of Chinese local pig breeds (Yang et al., 2003; Huang et al., 2020) and commercial pig breeds (Wang et al., 2021). There were many statistical methods to evaluate population genetic diversity, such as allele frequency and population heterozygosity. The higher the heterozygosity, the higher the genetic diversity. In this study, the MAF, Ho and He of the hybrid pig breed Yunan were higher than those of its founders Duroc and Huainan, indicating Yunan had a richer genetic diversity. Effective population size (Ne) is also an important indicator of diversity and species conservation. If the Ne was below 65, the breed might have a population crisis (Simon, 1999). The Ne of Yunan was 67. This suggested that although having experienced the threat of African swine fever, Yunan did not have a group crisis now.

ROH segments contains information about population inbreeding, and its length and frequency can reflect the population history. Compared with pedigree inbreeding number, the calculation of genomic inbreeding number based on ROH was more accurate and can better reflect the real inbreeding number of an individual (Purfield et al., 2012; Zanella et al., 2016; Deniskova et al., 2019; Bhati et al., 2020). A longer ROH usually indicates a closer genetic relationship while a shorter ROH indicates an ancient inbreeding. A larger number of ROHs and fragments means a higher probability of inbreeding (Kirin et al., 2010). Here, the average total length of ROH of Yunan was between Huainan and Duroc. Duroc had the highest average number of ROH per animal while Huainan had the lowest. Mean length of ROH was maximum in Yunan and minimum in Duroc. Duroc had the highest ROH based on inbreeding. F_*ROH*_ of Huainan and Yunan were lower than Duroc. Compared with some other local breed pigs (Mashen and Chun’an) in China (Cai et al., 2021; Dai et al., 2021) and European commercial breeds (Zhan et al., 2020), the average inbreeding coefficient of Yunan was relatively lower, and Yunan black pigs did not show obvious inbreeding.

From few large branches in NJ trees, population stratification in PCA, and having no one main lineage when K = 3 to 5 of ADMIXTURE implied much more breeding work should be done in Yunan. In addition, different subsets of Yunan may exist according to PCA, which was also indicated by t-SNE analysis. So, even if Yunan pigs had no risk of inbreeding and recession according to the population diversity and inbreeding analysis, but this diversity may partly be due to stratification within groups. Therefore, in the future breeding work of Yunan pigs, we should carry out mating among lineages according to the NJ molecular pedigree to avoid multiple invalid matings, otherwise the consistency of the population would not be improved.

Selection signals can reflect loci and genes that have been strongly selected in a population during long-term domestication. The selective signals we detected in Yunan black pigs were not only related to fat deposition, but also to growth. Both directions had both Duroc origin (Yunan-Duroc VS. Huainan) and Huainan origin (Yunan-Huainan VS. Duroc). For example, *GPRC6A* (Mukai et al., 2021) and *MBD5* (Du et al., 2012) for fatness and growth of Duroc while *ANGPTL3* (Jiang et al., 2018) and *DOCK3* (Reid et al., 2020) of Huainan. These results indicated that both Duroc and Huainan pigs had genetic variants affecting growth and fat deposition. In addition, some genes, such as *CDH13* (Göddeke et al., 2018) and *RAB23* (Hasan et al., 2020), were found to be involved in fat deposition and growth in the Yunan VS. Huainan-Duroc group. These loci might be derived from the founder effect of the initial breeding group of Yunan or might be the result of the 14 years of breeding process of Yunan.

Genomic loci associated with backfat thickness partially overlapped between different parities and different traits. This suggested the influence of some genes on backfat was a long-term process, there’s no time and space specificity. When it came to the nonoverlapped genes or regions, we could not come to a conclusion of time or space specificity. Because although the population used in this study covers the core population of Yunan black pigs, the number of populations was too small. This reminded us that under the epidemic environment, it was necessary to adopt multi-site conservation for species, especially for local genetic resources.

Three genes related to fat deposition, *ENPP2*, *EXT1* and *SLC30A8*, were found in the upper and lower 1 Mb of SSC4 (20.03–21.43 Mb) (Du et al., 2009; Hutley et al., 2011; Nishimura et al., 2014). Among the four linkage disequilibrium blocks in the 1.40 Mb interval of SSC4, only one gene, *SLC30A8*, was present in the fourth block (193.21 Kb). This gene is the star gene of type 2 diabetes. In 2009, there was a study that attempted to find the causal mutation for human type 2 diabetes by analyzing the correlation between the mutation of this gene with fat deposition in pigs (Du et al., 2009) but failed. Further analysis of this gene in Yunan might be needed.

In addition, QTL has been widely used in the study of important economic traits in pigs. The hunt for QTL in pigs has been ongoing for nearly 2 decades, beginning with the first publication of a QTL for fatness on pig chromosome 4 in 1994 (Andersson et al., 1994). In crossbreeding analysis, SNP markers and QTL linkage analysis can be used to identify genomic regions with complementary effects from potential resource groups, so as to increase the degree of genetic complementarity between varieties or lines in a planned way, and thus improve the economic traits of hybrids and the genetic diversity of varieties.

In conclusion, we analyzed genetic structure and mapped genomic regions affecting backfat thickness of Yunan black pigs. Although there was no risk of inbreeding depression, the stratification phenomenon existed in Yunan population. The increasing backfat thickness and decreasing consistency had a particular genetic basis. The 1.40 Mb interval of SSC4 (20.03–21.43 Mb) was a strong candidate region associated with backfat thickness. *ENPP2*, *EXT1,* and *SLC30A8*, particular *SLC30A8*, were strong candidate genes. Our findings are helpful for the subsequent breeding and conservation, as well as genomic improvement of backfat thickness of Yunan black pigs and suggested the importance of multi-site breeding conservation method.

## Data Availability

The datasets presented in this study can be found in online repositories. The names of the repository/repositories and accession number(s) can be found below: https://bigd.big.ac.cn, GVM000386.
